# The Multifaceted Role of Neuroprotective Plants in Alzheimer’s Disease Treatment

**DOI:** 10.3390/geriatrics7020024

**Published:** 2022-02-26

**Authors:** Tarek Zieneldien, Janice Kim, Chuanhai Cao

**Affiliations:** Department of Pharmaceutical Sciences, Taneja College of Pharmacy, University of South Florida, Tampa, FL 33620, USA; tarekz@mail.usf.edu (T.Z.); janicekim1@usf.edu (J.K.)

**Keywords:** Alzheimer’s disease, natural products, oxidative stress, herbal medicine, antioxidant, amyloid beta, tau protein, anti-inflammatory

## Abstract

Alzheimer’s disease (AD) is an age-related, progressive neurodegenerative disorder characterized by impaired cognition, memory loss, and altered personality. Many of the available pharmaceutical treatments do not alter the onset of disease progression. Recently, alternatives to developed drug candidates have been explored including medicinal plants and herbal treatments for the treatment of AD. This article examines the role of herbal plant extracts and the neuroprotective effects as alternative modes of intervention for AD progression. These extracts contain key metabolites that culminate alterations in AD progression. The traditional plant extracts explored in this article induce a variety of beneficial properties, including antioxidants, anti-inflammatory, and enhanced cognition, while also inducing activity on AD drug targets such as Aβ degradation. While these neuroprotective aspects for AD are relatively recent, there is great potential in the drug discovery aspect of these plant extracts for future use in AD treatment.

## 1. Introduction

Alzheimer’s disease (AD) is a heterogeneous neurodegenerative disorder currently affecting more than 6 million individuals in the United States and approximately 50 million individuals worldwide [1]. Furthermore, as a neurodegenerative disorder, AD irreversibly and gradually hinders cognition, memory, and the ability to carry out activities of daily living [2]. Although the etiology of AD has not been completely elucidated, there are various potentially causative factors that have been identified. In general, genetic factors have been associated with 5–10% of familial AD, with the remaining 90–95% being sporadic [3]. Furthermore, apolipoprotein E4 (ApoE4) homozygosity or heterozygosity has also been determined to significantly elevate the risk of AD development [4]. Nonetheless, age is considered the most critical risk factor for AD, with approximately 3% of individuals in the 65–74 age range and 17% of individuals in the 75–84 age range suffering from this disease [5,6,7]. Scientific efforts to discover a cure for AD have been expensive and disappointing, with current drugs mainly addressing the symptoms of the disorder and having limited effectiveness once the disease progresses in severity [8,9]. As such, despite the high prevalence of AD, only five drugs, consisting of galantamine, rivastigmine, donepezil, memantine, and a combination of donepezil and memantine, have been previously FDA-approved for its treatment [10]. Nonetheless, as of now, a new drug, aducanumab, has obtained accelerated approval [11].

The pathology of AD manifests with degeneration of the neurons, along with loss of synapses in the cortex, the hippocampus, and the subcortical structures [12]. In turn, this leads to the gross atrophy of the affected regions, yielding memory loss, inability to learn novel information, executive dysfunction, and an incapacity to fulfill daily living activities [13]. Furthermore, the pathological hallmarks of AD consist of neurofibrillary tangles and neuritic plaques, which are associated with cytoskeletal changes attributed to the hyperphosphorylation of microtubule-associated tau and the accumulation of Amyloid-beta in the brain [14]. The accumulation of Aβ has been suggested to prompt neurodegeneration, thereby leading to the clinical dementia often witnessed in AD patients [15].

In traditional medicine, herbal remedies have often been utilized for health promotion and treatment [16]. However, there has been minimal scientific attention in the usage of these traditional medications. The bioactive components present in traditional herbs help alleviate a variety of AD symptoms including poor cognition and memory loss. These components provide neuroprotection and improved memory through their mechanisms of action [17]. This review provides an overview of herb-derived therapeutics for AD that relieve the symptoms. 

## 2. AD Pathological Mechanisms

AD is a progressive age-related neurodegenerative disorder that is responsible for most cases of dementia [5]. Besides the environmental and genetic factors, which are presupposed to contribute to AD etiology, there are numerous hypotheses which have been elucidated to attempt to explicate AD. The most prevalent hypotheses are the Aβ cascade hypothesis, the inflammation hypothesis, the cholinergic hypothesis, the tau hypothesis, and the oxidative hypothesis [18,19,20]. The Aβ cascade hypothesis implicates Aβ peptides as the AD causative agent due to the extracellular deposition of the Aβ peptides as senile plaques, with the neurofibrillary tangles resulting in neuronal loss, dementia, and vascular damage [21]. Neurofribrillary tangles are also considered an AD hallmark and principally consist of tau, which is a microtubule-associated scaffold protein [22]. The aggregation of tau impairs the axons, leading to neurodegeneration [23]. Currently, the inflammation hypothesis has gained prominence as one of the major AD pathological factors, encompassing the immune response sustained in the brain [24]. Continuous activation of the brain’s immune cells, such as microglia, leads to the release and production of various proinflammatory cytokines, which aids tau and Aβ pathologies and leads to the loss of neurons [24]. Damage to the cholinergic neurons has also been accepted as a critical pathological chance associated with AD-related cognitive impairment [25]. As such, the cholinergic hypothesis proposes that cholinergic neuronal dysfunction in the brain could substantially contribute to the AD-related cognitive decline [25]. In general, this hypothesis is supported by the usage of cholinesterase inhibitors in the treatment of AD [26]. Furthermore, oxidative stress has also been determined to have a crucial role in AD pathogenesis [27]. In fact, a vast amount of evidence indicates that AD is perpetually accompanied by elevated oxidative cellular stress in the brain, yielded by the elevated production of free radicals, reduced polyunsaturated fatty acid, elevated protein and DNA oxidation, elevated lipid peroxidation, and the aggregation and accumulation of Aβ, which cause oxidative stress [28].

## 3. Herbal Neuroprotective Strategies

Currently, there is a vast amount of evidence suggesting that the primary pathological causative factor of AD is Aβ accumulation [29]. As such, reducing the amount of Aβ has been a major target in the development of AD therapeutics [30,31,32]. Nonetheless, effective therapeutic regimens for AD may necessitate the utilization of numerous neuroprotective agents [33,34]. As of now, various molecular targets have been recognized as mediating pathophysiological processes [35]. The identification of these targets could potentially allow the development of high-yield neuroprotective techniques for AD treatment [35]. Potential neuroprotective mechanisms fixate on inhibiting deleterious intraneuronal pathways triggered by toxic stimuli and Aβ via the interaction with neuronal targets [36]. Popular neuroprotective strategies for AD management consist of discovering small molecules with the ability to block these Aβ interactions with intracellular and extracellular targets to avoid caspase activation and the expression of proapoptotic proteins, to reduce the stress kinase signaling cascades, to inhibit tau protein phosphorylation, to counterpoise cholinergic functional loss, and to boost neuronal plasticity while blocking excitotoxicity [37,38,39,40,41]. In general, natural products are advantageous because some have the ability to exhibit their neuroprotective effects through a plethora of differing approaches. As such, herbal medicine for AD could be multidimensional when the herb being utilized has various effective bioactive compounds [42]. Additionally, the synergistic action of herbal extracts and mixtures also has the potential to eliminate severe adverse effects correlated with the utilization of a single xenobiotic product, allowing for a broader spectrum of activity and reducing the chances of pathogenic resistance [43].

## 4. Herbal Neuroprotective Effects

Numerous natural bioactive compounds have been demonstrated to have neuroprotective roles via antioxidation, antineuroinflammatory, anti- Aβ and tau aggregation, as well as targeting cholinergic neurotransmission [44,45,46]. As of now, it is reasonable to presume that AD onset and progression could be delayed or slowed down via the utilization of neuroprotective natural products that elicit their effects by affecting various pathological targets [47]. Moreover, herbal administration and combinations for medical usage has been widely utilized in traditional Chinese medicine, Native American medicine, and Ayurveda [48,49]. For AD, a wide variety of plant extracts has been utilized and tested in clinical studies (Table 1).

*Withania somnifera*, common name ashwagandha, is an evergreen shrub in the Solanaceae family [78,79]. *W. somnifera* is one of the most conspicuous herbs prescribed for AD [80,81]. In general, it is prescribed as a nerve tonic and energy booster [81]. *W. somnifera,* as an adaptogen, has been demonstrated to possess free radical scavenging and antioxidant and immune-boosting activities [78]. *W. somnifera* contains a plethora of bioactive compounds of medical interest, such as withanolides A-Y, withanone, withasomniferols A-C, dehydrowithanolide-R, withasomidienone, and other ergostane-type steroidal lactones [82,83]. The plant also includes alkaloids, beta-sitosterol, and phytosterols sitoindosides VII-X [83]. Some of these constituents have been demonstrated to scavenge free radicals produced during AD pathological progression [83,84]. Molecular modeling research studies have elucidated that withanamides A and C have the ability to bind to the active motif of Abeta-25-35 and avert the formation of fibrils [85]. Subsequently, these compounds have been shown to protect neuronal rat cells and PC-12 cells from Beta-amyloid-induced neuronal death [51,86]. Consequently, therapeutic treatments that consist of *W. somnifera’s* methanol extractions have been shown to trigger the outgrowth of neurites in a time- and dose-dependent approach in human neuroblastoma cells [87]. Another research study consisting of cultured cortical rat neurons displayed a diminishment of pre- and postsynaptic stimuli, as well as dendritic and axonal atrophy, when treated with Aβ peptides [88]. Consequent therapeutic treatment with withanolide A displayed significant regenerative properties of dendrites and axons, and even displayed restorative properties of the pre- and postsynapses in the cultured neurons [88]. 

Withanolide A has been shown to inhibit Abeta25-35-induced axonal and dendritic degeneration in the hippocampus and cerebral cortex, while also seemingly restoring synapses and Aβ -peptide-induced memory impairment in mice [89]. Additionally, the ameliorative outcomes were retained after treatment discontinuation [89]. Subsequently, aqueous extracts of *W. somnifera* elevated the activity of choline acetyl transferase and acetylcholine in rats, potentially explicating the memory- and cognition-enhancing effects [87,90]. Consequently, root extract treatments led to low-density lipoprotein receptor-related protein upregulation, thereby boosting Aβ clearance and ameliorating AD pathology in APP/PS1 mice [91]. Similarly, administering semipurified extracts of *W. somnifera* orally inhibited Aβ peptide accumulation and reversed behavioral impairment in APP/PS1 mice models of AD [91]. These ameliorative effects were mediated via the enhancement of liver low-density lipoprotein receptor-related protein. Consequently, Drosophila melanogaster models of AD have also elucidated that *W. somnifera* treatment could mitigate Abeta toxicity while seemingly boosting longevity [92]. Nonetheless, although a vast amount of literature has reported the ameliorative therapeutic effects of *W. somnifera*, clinical data related to its use for cognitive impairment are limited [93].

In a double-blind, randomized, placebo-controlled study encompassing 50 subjects with mild cognitive impairment (MCI), the subjects were treated with 300 mg of *W. somnifera* root extracts twice daily or with a placebo for eight continuous weeks [94]. At the end of the eight weeks, the *W. somnifera*-treated group showed significant enhancements in information-processing speed, attention span, and executive function [94]. As such, these two studies provide evidence for *W. somnifera’s* enhancing roles in memory and executive function in subjects with MCI [91,94].

*Bacopa monnieri,* common names waterhyssop or brahmi, is a perennial plant in the Plantaginaceae family [95]. *B. monnieri* is a nootropic herb with low toxicity and has been traditionally utilized as a memory booster and neural tonic in a plethora of ailments [95]. There have been studies that have elucidated evidence for the role of *B. monnieri* in epilepsy, dementia, and Parkinson’s disease attenuation [96,97]. Furthermore, it has also been utilized for stress, asthma, and insomnia [98,99]. The bioactive phytochemicals present in *B. monnieri* encompass sterols, polyphenols, sulfhydryl compounds, saponins, bacosides A and B, betulic acid, bacopasides II, IV, and V, and bacosaponins A, B, C, D, and E [100]. The neuroprotective activity of *B. monnieri* could potentially be due to the bioactivity of the phytochemicals, thereby explaining its usage in traditional medicine. In fact, research studies conducted in vitro and in vivo demonstrate that these phytochemicals contain free radical scavenging and antioxidant activities via the blockage of lipid peroxidation in various brain areas [57,101]. *B. monnieri* elicits its role via the reduction in divalent metals, reduction in lipid peroxide formation, inhibition of lipoxygenase, and through its scavenging activity of reactive oxygen species [102].

Various studies have seemingly elucidated the role of *B. monnieri* in terms of intellect and memory [103,104]. In order to illuminate the neuroprotective activities of *B. monnieri* in rat models of AD, researchers conducted studies examining the administration of 20, 40, and 80 mg/kg alcoholic extracts of *B. monnieri* on rats for 2 weeks prior to and 1 week after intracerebroventricular ethylcholine aziridinium ion administration [56]. The Morris water maze was utilized to test spatial memory, and histological assays were utilized to assess the density of cholinergic neurons [56]. In this case, the *B. monnieri* extract appeared to enhance the escape latency time in the Morris water maze and blocked the diminishment of cholinergic neuron density [56]. Another conducted study demonstrated the backtracking of colchine-induced cognitive impairments by *B. monnieri* extracts [105]. Similarly, *B. monnieri* extracts attenuated oxidative damage caused by colchicine via the reduction in the protein carbonyl content while also restoring antioxidant enzyme activity [105].

The majority of the research studies examining the cognitive enhancement elicited by *B. monnieri* in humans have been centered on normal geriatric individuals. In a randomized, double-blind, placebo-controlled clinical trial encompassing 35 subjects of 55 years and older, the subjects were administered a 125 mg dosage of *B. monnieri* extract or a placebo twice a day for an interval of 12 weeks, with a placebo period that consisted of an additional four weeks [58]. The researchers conducted numerous memory tests focused on logical memory, visual reproduction, paired-association learning, general information, orientation, digit forward, and digit backward subtests [68]. The subjects were then given a score on each subtest, with the total memory consisting of the result of all of the subtests [58]. Treatment with *B. monnieri* extract significantly improved paired-association learning, mental control, and logical memory in the subjects in comparison to the placebo groups at 8 and 12 weeks following trial initiation [58]. These results propose that *B. monnieri* extracts could be beneficial in age-associated memory deficit treatment. 

In another study conducted by Dimpel et al., ten subjects were administered 500 mg of *Sideritis scardica* extract, 320 mg of *B. monnieri* extract, or a mixture via the utilization of a crossover design [106]. *S. scardica* extracts, which are abundant in a plethora of flavonoids, have been demonstrated to inhibit aggregation and toxicity of amyloid-beta in *Caenorhabditis elegans* models of AD [107]. Consequently, the d2-concentration test was utilized to characterize thinking, performance, tension with selective orientation of perception, and wakefulness of the subjects [106]. The results of the treatment group elucidate that *S. scardica* extract yielded improved d2-concentration tests scores when combined with low-dose *B. monnieri* extract. Likewise, *B. monnieri* alone exhibited enhanced effects after continuous treatment, proposing repetitive dosing of *B. monnieri* as a potential therapeutic alternative for subjects with MCI. 

Additionally, another multicenter, prospective, and noncomparative clinical trial encompassing 104 MCI subjects and a duration of 60 days was carried out [108]. The subjects were administered *B. monnieri* in combination with vitamin E, phosphatidylserine, and astaxanthin [108]. Mnemonic and cognitive performances were tested with the clock-drawing test and the Alzheimer’s Disease Assessment Scale–Cognitive Subscale (ADAS-cog), which have the ability to assess the risk of MCI-to-AD advancement [108,109]. The researchers noticed significant enhancement in the clock-drawing test and the Alzheimer’s Disease Assessment Scale–Cognitive Subscale after 60 days when compared to the baseline of the subjects [108]. Although memory is greatly affected by a plethora of factors, such as hormones, cyclic AMP, neurotransmitters, focus, attention, synapse formation, nutrients, and protein transcription, a couple of these processes could potentially be modulated by *B. monnieri* in isolation or in combination with other compounds as partially shown in these studies [56,57,101,110,111].

*Ginkgo biloba*, common name gingko or maidenhair tree, is a tree species in the Ginkgoaceae family native to China [112]. The leaf extract of *G. biloba* has been prominently utilized for its ability to enhance memory and deter age-related impairments [113]. *G. biloba* contains terpenoids and flavonoids, which are considered the main pharmacologically active constituents [114]. The majority of the clinical trials conducted utilizing *G. biloba* extract included a combination of ginkgolic acids, terpene lactones, and flavonoid glycosides [115,116,117]. *G. biloba* extract has demonstrated ameliorative effects in AD, tinnitus, and cancer treatments [117,118,119]. The proposed mechanisms of *G. biloba* extract include Beta-amyloid peptide aggregation inhibitory effects, antioxidant activities, antiplatelet-activating factor activity, and reduced expression of the peripheral benzodiazepine receptor, which is helpful for stress relief [120,121,122].

*G. biloba* extract has been shown to reverse nitric oxide and Beta-amyloid toxicity in vitro while simultaneously decreasing apoptosis in vivo and in vitro [120,123,124]. Therapeutic treatment with *G. biloba* extract has also shown the ability to improve spatial learning and memory of aluminum-intoxicated rats, potentially due to a reduced APP expression and caspase-3 in the brains of treated-rats [125]. Modest cognitive function enhancement was noted in AD subjects in numerous randomized, placebo-controlled, double-blind clinical trials [126,127]. Furthermore, a meta-analysis was conducted by Hashiguchi et al. examining the effects of *G. biloba* treatment in nine studies (seven of which utilized the Syndrome Kurztest and ADAS-Cog) at doses of 120 mg with a 26-week duration for efficacy parameters [128]. Their meta-analysis showed that the standard mean differences in seven of the studies favored *G. biloba* treatment over the administered placebo, although two of the studies did not show statistically significant differences between *G. biloba* and placebo treatment in ADAS-Cog [128]. In the AD and vascular dementia subgroups, the standard mean differences in the Syndrome Kurztest favored *G. biloba* over placebo treatment, with 240 mg daily dosages having significantly enhanced standard mean differences in the Syndrome Kurztest [128].

*Centella asiatica,* common names Asiatic pennyworth, Indian pennyworth, Gotu kola, and kodavan, is a perennial plant in the Apiaceae family [129]. *C. asiatica* is native to Asian wetlands and has been utilized as a medicinal herb for cogniceutical and nutraceutical purposes [129]. *C. asiatica* is presupposed to heal skin, boost kidney and liver health, and strengthen the brain [130]. *C. asiatica* is also considered to be a rejuvenating plant for brain and nerve cells, since it is thought to augment intelligence and enhance memory [131]. In vitro research studies utilizing madasiatic acid, madecassoside, asiatic acid, and asiaticosides *C. asiatica* derivatives have elucidated that these compounds have the ability to block hydrogen peroxide and beta-amyloid-induced cellular death, as well as to reduce the concentration of free radicals, thereby proposing a possible role for *C. asiatica* in AD treatment and prevention [64,132,133].

Ethanolic extracts of *C. asiatica* have also elicited neurite outgrowths in SH-SY5Y human cells in the presence of the nerve growth factor and expedited regeneration of the axons in rats [63]. *C. asiatica* leaf extracts also demonstrated enhanced memory and learning in rats via the modulation of various neurotransmitters in rat brains, inclusive of noradrenaline, 5-hydroxytryptamine, and dopamine [134]. This advocated for the possible therapeutic role of *C. asiatica* in the therapeutic treatment of AD-associated cognitive decline. 

Furthermore, by utilizing double-transgenic PS/APP mice, which spontaneously generate Aβ plaques, studies have demonstrated that treatment with 2.5 mg/kg of *C. asiatica* extract significantly reduced hippocampal Abeta1-40 and Abeta-1-42 levels [65]. Consequently, long-term treatment with higher dosages of aqueous *C. asiatica* extract eventuated in a significant decline in Congo red-positive amyloid fibrillar plaques [65]. Additionally, there was significant scavenging activity of reactive oxygen species with the lowest dosage utilized, which was 25 μg, and 83% of the reactive oxygen species were removed with a 250 μg dosage [65].

Various asiatic acid derivatives, as some of the most common phytochemicals found in *C. asiatica*, demonstrated significant cognitive-enhancing activities when utilizing a scopolamine-induced model of memory impairment [135]. Scopolamine was utilized because it yields transient memory deficiencies analogous to the early stages of AD [135]. Utilizing the Morris water maze test and passive avoidance, the study demonstrated that pretreatment with three differing asiatic acid derivatives significantly enhanced memory in comparison to the scopolamine-treated mice that were not given any derivatives [135]. The improvement in cognition witnessed in the treated mice was attributed to elevated choline acetyltransferase activity, yielding enhanced acetylcholine synthesis [135].

Subsequently, in a double-blind, randomized, placebo-controlled research study, *C. asiatica* extract was administered twice a day for two months to 28 healthy subjects at 250, 500, and 750 mg dosages [136]. Mood and cognitive performance were noted at baseline, after the first administration, after one month, and after two months post-treatment [136]. The experimental results elucidate that the highest dosage indeed enhanced working memory, and improvements in self-rated mood were also noted post-treatment, implying that *C. asiatica* could be helpful in mitigating age-associated cognitive impairment while also boosting mood in healthy geriatric subjects [136].

*Crocus sativus*, also known as autumn crocus or saffron crocus, is a flowing plant in the Iridaceae family [137]. It is prominent for producing the crimson-colored spice saffron from the filaments that grow within the flower [138]. Saffron is widely cultivated in various countries, such as Greece, Iran, and India [139]. Furthermore, the spice has a wide variety of applications in the cosmetic, textile, and medicinal industries [139,140]. Safranal, a carboxaldehyde, is a major constituent of saffron [68]. In vivo and in vitro research studies have demonstrated that the phytochemicals found in saffron contain antiamyloidogenic, antioxidant, and anti-inflammatory properties [67,68].

In order to examine saffron’s efficacy in therapeutic treatment of mild to moderate AD, a double-blind clinical study enrolled 46 subjects that were randomly assigned to obtain 30 mg/day of saffron or a placebo [141]. After 16 weeks, saffron significantly improved subject outcomes in cognitive performance compared to the placebo when observing their Alzheimer’s Disease Assessment Scale–Cognitive Subscale scores [141]. Additionally, there were no significant differences in the treatment or placebo-controlled groups in terms of adverse effects reported [141]. Subsequent to this study, researchers distinguished donepezil, the cholinesterase inhibitor, with a saffron extract, in subjects suffering mild to moderate AD [69]. In a double-blind, randomized, controlled clinical trial with a duration of 22 weeks, 54 Persian-speaking subjects were randomly dispensed 10 mg/day of donepezil or 30 mg/day of saffron [69]. After the conclusion of the study, it was determined that saffron had similar effects in enhancing cognitive function and less side effects in comparison to donepezil [69]. The researchers proposed that saffron’s ability to treat mild to moderate AD could potentially be due to its inhibitory activity towards deposition and aggregation of beta-amyloid plaques [69].

A pilot study examining efficacy and safety compared memantine with saffron extract in terms of cognitive defect reductions [142]. A total of 68 subjects with moderate to severe AD were recruited and enrolled in a double-blind, randomized, parallel-group research study [142]. The subjects were administered 20 mg/day of memantine or 30 mg/day of saffron extract capsules for 12 months [142]. The saffron extract was comparable with memantine in decreasing cognitive decline and had a seemingly low adverse effect rate in subjects with moderate to severe AD [142]. Due to these studies, saffron could be considered an herbal spice with the ability to enhance the completing activities of daily living as well as boosting cognitive functions in subjects with MCI and AD [69,141,142]. Subsequently, the studies demonstrated no severe or prominent adverse effects, making saffron a suitable natural therapeutic for these patients. 

*Curcuma longa*, or turmeric, is a flowering plant native to Southeast Asia and the Indian subcontinent in the Zingiberaceae family [143]. The curcuminoid polyphenolic compounds in this rhizome plant lead to the display of the bright orange-yellow color [144]. In general, turmeric is antibacterial, antiseptic, and anti-inflammatory and has a long history of medicinal usage for allergies, boosting immunity, stimulating digestion, preventing infection, boosting liver detoxification, and balancing cholesterol levels [144,145,146]. The bioactive components of turmeric consist of water-soluble curcuminoids and turmerone oil [147]. The curcuminoids encompass cyclocurcumin, bisdemethoxycurcumin, demethoxycurcumin, and curcumin [148]. Moreover, curcumin is the main curcuminoid associated with reduced AD risk due to its anti-inflammatory activity [149]. In vitro studies have elucidated that curcumin has the ability to neutralize reactive oxygen species and block lipid peroxidation, with curcumin being determined to have a greater potency than vitamin E [150,151].

Administering curcumin orally to aged mice with advanced deposits of plaque yielded a significant diminishment in the plaque loads [152]. Additionally, curcumin was also associated with reduced inflammation, amyloid pathology, and oxidative damage in mice models of AD [152,153]. Likewise, administering curcumin via peripheral injections crossed the blood–brain barrier and decreased plaque levels and blocked subsequent plaque development [152]. Furthermore, various studies using animal models of AD have demonstrated enhanced cognitive function in curcumin-treated groups, potentially due to curcumin’s ability to decrease the levels of Aβ plaques, as well as its antioxidant and anti-inflammatory properties [154,155]. To illustrate this, a double-transgenic APP/PS1 AD model allowed researchers to inspect the effects of two different curcumin dosages, including a 160 ppm low dose and 1000 ppm high dose for a period of 6 months [156]. Both doses reported significant cognitive enhancement, but the higher curcumin dosage generated better cognitive improvement [156]. Likewise, curcumin decreased the deposits of Aβ, potentially via autophagy promotion [156]. Nonetheless, due to curcumin’s inefficient penetration of the BBB, nominal bioavailability, and quick gastrointestinal metabolism, various curcumin analogs have been tested [157,158,159]. Even though the derivatives provided differing improved outcomes based on the disease model, they were all effective at decreasing plaque-associated pathology and enhancing cognitive function [160,161,162].

Curcumin also has the ability to reverse cognitive deficits in numerous animal models of AD [163,164,165]. Regardless of the administration route, higher dosages have been determined to be more efficacious than lower dosages, and cognitive enhancement was more prominent when curcumin was administered with piperine, which has a plethora of pharmacological and positive health effects against chronic disorders [146]. Subsequently, there is evidence suggesting that metals similar to iron, zinc, or copper may have a role in AD-related pathogenesis [166]. The metals are found concentrated in AD-affected brains and trigger oxidative neurotoxicity and amyloid aggregation [167]. Curcumin has been shown to produce strong metal complexes, thereby blocking mentally induced Aβ inflammation, toxicity, and aggregation [163,168].

Nonetheless, clinical studies conducted utilizing curcumin have been limited in number. Although research has demonstrated that curcumin in combination with vitamin C, B vitamins, piperine, and other dietary supplements has neuroprotective and synergistic effects, further research is necessary to conclusively state curcumin’s ability at ameliorating cognitive function in humans [146,162,169]. As such, if future studies elevate curcumin’s systemic bioavailability and enhance BBB penetration, curcumin could be a promising therapeutic for AD. 

Fuzhisan, a Chinese herbal complex comprising *Anemone altaica* in the Araceae family, *Glycyrrhiza uralensis* in the Leguminosae family, *Scutellaria baicalensis* in the Labiatae family, and Ginseng root in the Araliaceae family, has been clinically utilized for senile dementia [75]. Fuzhisan’s neuroprotective effects have been proposed to be associated with anti- Aβ accumulation and anti-apoptotic effects, as well as elevating acetylcholine levels and enhancing neurotropic effects [170]. Consequently, studies have shown that Fuzhisan inhibits Abeta25-35-induced neurotoxicity, with the induction of the ADAM10 and SIRT1-Fox0 pathway serving a potential role in neuroprotection, since PC12 cells treated with Fuzhisan demonstrated significantly elevated levels of ADAM10 [170]. Patel et al. elucidated the protective effects of SIRT1 on AD, showing that caloric restrictions diminished plaque formation and Aβ levels in the brains of AD transgenic mice models [171]. Consequently, deficiencies in SIRT1 have also been correlated with enhanced levels of phosphorylated tau in the neurons and the number of neurofibrillary tangles in the brains of AD subjects [171,172]. In an in vivo study in aged rats, Fuzhisan administered at 0.3, 0.6, and 1.2 g/kg/d ameliorated cognitive function. Western blot and spectrophotometry results demonstrate that Fuzhisan elevated the production and activity of choline O-acetyltransferase and acetylcholine contents in the hippocampus [75]. Furthermore, a research study conducted by Bi et al. assessed the glucose metabolism and cognitive performance by ADAS-Cog in 22 patients, with 12 receiving 10 mg/day of Fuzhisan and 10 receiving a placebo [76]. These studies, especially the randomized, placebo controlled human trials suggest that various herbal products could potentially ameliorate cognitive function (Table 2).

## 5. Medicinal Plants for AD with Limited Studies

Currently, there are a plethora of other medicinal plants that show preventative and therapeutic activity for AD [174]. Nonetheless, in vitro and in vivo studies are limited, with most data deriving from observational studies. These plants include *Commiphora wightii*, *Tinospora cordifolia*, *Hypericum perforatum*, *Rhodiola rosea*, *Moringa oleifera*, *Convolvolus pluricaulis*, *Hericium erinaceus*, *Camellia sinensis,* and others [174,175,176,177,178,179]. Similarly, there are also neuroprotective natural products that could be obtained from food. To illustrate this, *Allium sativum* in the Alliaceae family has shown to be a potent anti-neuroinflammatory, antioxidant, and regulator of neurotransmitter signaling when referring to aged garlic extract, which could ameliorate AD pathogenesis [180,181]. Likewise, juice and extracts from *Punica granatum* have also shown neuroprotective effects in animal models, potentially by counteracting oxidative damage, minimizing inflammation of the brain and soluble Aβ 42 and hippocampal amyloid deposition [182,183].

Consequently, natural products, such as cannabidiol (CBD) and tetrahydrocannabinol (THC), have also shown to be effective in vitro and in vivo [184,185,186,187]. In PC12 neuronal cells, CBD has shown protection against oxidative stress, Aβ-induced neurotoxicity, inhibition of tau hyperphosphorylation, prevention of proinflammatory gene transcription, and inhibition of Aβ-induced tau hyperphosphorylation [188,189]. In vivo, CBD has shown attenuation of Aβ-induced neuroinflammatory responses by minimizing proinflammatory gene and mediator expression, as well as minimized reactive gliosis [187,190]. In vivo studies using CBD and THC have shown enhanced memory in the active avoidance and two-object recognition tasks [186]. Additionally, it has been shown to reduce soluble Abeta42 levels and alter the plaque composition [186].

## 6. Future Directions in Herbal Medicine

Currently, approximately 6.2 million Americans aged 65+ are suffering from Alzheimer’s dementia [1]. Additionally, that number is expected to grow to 13.8 million Americans by 2060 if effective therapeutics are not developed and utilized to halt, prevent, or slow AD-related pathogenesis [1]. Consequently, AD has an astoundingly high economic burden, with an approximated USD 305 billion having been spent on AD treatment in 2020 [191]. As such, finding effective therapeutic treatments are necessary to enhance patient outcomes. Even though various novel approaches have been found for symptomatic treatment and numerous disease-modifying therapies are under development, most clinical trials related to AD have not been successful [192]. Due to this, a significant deviation from monotherapeutic treatments has taken place to favor multitherapeutic, individualized, and comprehensive approaches since AD is a highly heterogeneous disorder [193,194].

Tau and Aβ have been shown to boost the loss of blood–brain–barrier (BBB) integrity [195]. Thus, a critical challenge in AD drug delivery relies on circumventing the BBB, which averts the entry of a plethora of possible therapeutic agents [195]. In general, the most common administration route is oral, but it has not been clearly elucidated whether herbal components can access the central nervous system from systemic circulation. Furthermore, rapid metabolism, limited solubility in aqueous environment, and incomplete distribution in the CNS are further limits that must be overcome. Thus, intranasal administration is an efficient and noninvasive administration route that could bypass the BBB and directly target the central nervous system [196]. Utilizing this method of delivery, herbs in medicated oil or dry powder forms could be administered directly to the subject. Medicated oils could also contain a mixture of lipid-soluble and lipophilic molecules to warrant the synergistic interactions between the varied herbal components. The benefits of intranasal delivery comprise brain injury avoidance, surmounting the requirement of implanting delivery devises, and reducing the systemic-administration-associated side effects [196,197]. Utilizing the intranasal administration technique, researchers have successfully treated memory loss in transgenic mice models of AD [198]. However, this method also has some limitations, such as a particularly small volume of administrated drugs, the limited surface area in the olfactory epithelium, and a relatively short retention time for drug absorption [199,200]. Thus, further research is required to support and enhance the usage of intranasal administration for herbal delivery.

In general, a large amount of evidence has shown that various herbs and natural bioactive products could be effective in AD treatment while having minimal severe adverse effects [201]. Even though it is not fully understood, the AD pathological process is proposed to be multifactorial [202]. As such, neuroprotective techniques encompassing a plethora of mechanisms of action are crucial for AD treatment and prevention. Natural product extracts and mixtures, with various bioactive compounds and neuroprotective mechanisms, are advantageous in drug discovery for AD. However, more research is necessary to address the concerns associated with herbal medicine and natural medicine for AD. Consequently, although the use of some herbs, such as those of *C. longa* and *B. monnieri,* have demonstrated slight clinical improvements, many natural products such as polyphenols remain of interest in the treatment of neurodegenerative disorders [203]. Additionally, some limitations include chemical instability, since curcumin and resveratrol are chemically unstable [204]. Likewise, low bioavailability, such as that observed with curcumin, is also a major issue [157]. Therefore, it has been challenging to translate effective preclinical results. Nonetheless, numerous studies have attempted to improve bioavailability by employing nanocarriers and nanotechnology, which could potentially enhance clinical efficacy and therapeutic response [205]. One notable example has been that of nanolipid epigallocatechin-3-gallate particles, which have demonstrated the ability to increase α-secretase, enhancing their ability in vitro and increasing epigallocatechin-3-gallate’s oral bioavailability by greater than two-fold [206]. As such, more comprehensive quality control and practical guidelines, in addition to novel strategies and approaches to promote central nervous system access of the herbal neuroprotective agents, could potentially allow natural product therapy to play an essential role in AD preventative and therapeutic measures.

## Figures and Tables

**Table 1:** The neuroprotective effects of herbs administrated for AD treatment. Neuroprotective herbs act via various mechanisms to elicit their ameliorative effects on AD patients. The neurotherapeutic effects of the herbs aid in the restoration and enhancement of memory and cognitive function.

Herb	Neuroprotective Effect	Type of Research Study	References
*Withania somnifera*	Anti-inflammatory, antioxidant, inhibits abeta production and neurite outgrowths, boosts neural regeneration, reverses dysfunction of the mitochondria, enhances processing speed, social cognition, auditory–verbal working memory	In vitro, in vivo, clinical research studies	[50,51,52,53,54]
*Bacopa monnieri*	Anti-inflammatory, enhances attention, memory, executive function, inhibits abeta production, enhances cardiac function, antioxidant	In vitro, in vivo, clinical research studies	[55,56,57,58]
*Gingko biloba*	Enhances mitochondrial function, antioxidant, boosts neurogenesis, stimulates cerebral blood flow	In vitro, pre-clinical and clinical research studies	[59,60,61,62]
*Centella asiatica*	Decreases oxidative stress, apoptosis, abeta levels, boosts dendritic growth, enhances memory and improves mood	In vitro, in vivo, clinical research studies	[63,64,65,66]
*Crocus sativus*	Antiamyloidogenic, antidepressant, neuroprotective effects, immune system modulation, antioxidant	In vitro, in vivo, clinical research studies	[67,68,69,70]
*Curcuma longa*	Anti-inflammatory, antioxidant, antimicrobial, inhibits abeta production, anti-apoptosis	In vitro, in vivo, pre-clinical and clinical research studies	[71,72,73,74]
Fuzhisan (*Anemone altaica*, *Glycyrrhiza uralensis*, *Scutellaria baicalensis*, *Panax ginseng*)	Anti-abeta accumulation, antiapoptosis, neurotropic effects, and enhances acetylcholine levels	in vitro, in vivo, clinical research studies	[75,76,77]

**Table 2:** Neurotherapeutic effects of *Withania somnifera, Bacopa monnieri, Gingko biloba, Centella asiatica, Crocus sativus, Curcuma longa, and* Fuzhisan in randomized, placebo controlled human trials. In these trials, no severe adverse effects were noted for any of the administered extracts or natural compounds.

Herb	Conducted By	Study Design	Sample Size	Dosage Regimen	Metrics Tested	Findings
*Withania somnifera*	Pingali et al. [53]	Randomized, placebo controlled, double-blind study	20	250 mg twice daily for 14 days	Reaction time, choice discrimination, digit symbol substitution, digit vigilance, card sorting tests, finger-tapping test	Significant improvement in reaction time, choice discrimination, digit vigilance, card sorting tests, and digit symbol substation No effect in finger-tapping test
*Bacopa* *monnieri*	Raghav et al. [58]	Randomized, placebo controlled, double-blind study	40	125 mg twice daily for 12 weeks, followed by 4 weeks of placebo (16-week total duration)	Mental control, logical memory, digit forward, digit backward, visual reproduction and paired-associate learning	Significant improvement in mental control, logical memory, and paired-associate learning
*Gingko* *biloba*	Le Bars et al. [126]	Randomized, placebo controlled, double-blind study	309	120 mg once daily for 52 weeks	ADAS-Cog	Modest improvement in cognitive performance measured by ADAS-Cog and noted by caregivers
*Centella* *asiatica*	Wattanathorn et al. [136]	Randomized, placebo controlled, double-blind study	28	250, 500, and 750 mg once daily for 8 weeks	Bond–Lader mood scale, alert factor, content factor, calm factor, word recognition, image recognition	Increased accuracy in word recognition, image recognition, alertness, and calmness, with high dosage showing greater effects. No significant difference in content factor between treatment and placebo groups.
*Crocus* *sativus*	Akhondzadeh et al. [69]	Randomized, placebo controlled, double-blind study	22	15 mg twice daily for 22 weeks	ADAS-Cog	Effectively similar to 10 mg daily of donepezil in the ADAS-Cog scale
*Curcuma longa*	Baum et al. [173]	Randomized, placebo controlled, double-blind study	34	1000 to 4000 mg once daily for 26 weeks	ADAS-Cog	No cognitive decline in enrolled subjects during study duration
Fuzhisan (*Anemone altaica*, *Glycyrrhiza uralensis*, *Scutellaria baicalensis*, *Panax ginseng*)	Bi et al. [76]	Randomized, placebo controlled, double-blind study	22	10 mg once daily for 12 weeks	ADAS-Cog, neuropsychiatric index	Significantly improved ADAS-Cog and neuropsychiatric index scores compared to placebo

## Data Availability

Not applicable.
